# Effect of Staurosporine in the Morphology and Viability of Cerebellar Astrocytes: Role of Reactive Oxygen Species and NADPH Oxidase

**DOI:** 10.1155/2014/678371

**Published:** 2014-08-17

**Authors:** Mauricio Olguín-Albuerne, Guadalupe Domínguez, Julio Morán

**Affiliations:** División de Neurociencias, Instituto de Fisiología Celular, Universidad Nacional Autónoma de México, Apartado Postal 70-253, 04510 México, DF, Mexico

## Abstract

Cell death implies morphological changes that may contribute to the progression of this process. In astrocytes, the mechanisms involving the cytoskeletal changes during cell death are not well explored. Although NADPH oxidase (NOX) has been described as being a critical factor in the production of ROS, not much information is available about the participation of NOX-derived ROS in the cell death of astrocytes and their role in the alterations of the cytoskeleton during the death of astrocytes. In this study, we have evaluated the participation of ROS in the death of cultured cerebellar astrocytes using staurosporine (St) as death inductor. We found that astrocytes express NOX1, NOX2, and NOX4. Also, St induced an early ROS production and NOX activation that participate in the death of astrocytes. These findings suggest that ROS produced by St is generated through NOX1 and NOX4. Finally, we showed that the reorganization of tubulin and actin induced by St is ROS independent and that St did not change the level of expression of these cytoskeletal proteins. We conclude that ROS produced by a NOX is required for cell death in astrocytes, but not for the morphological alterations induced by St.

## 1. Introduction

Apoptotic cell death plays a key role in the shaping of the nervous system during development and in the etiology and progression of certain neurodegenerative disorders [1, 2]. Apoptosis is a highly regulated process that involves several morphological alterations, including cell shrinkage and chromatin condensation. These morphological changes are accompanied by a number of biochemical changes [3], including the activation of a group of proteases known as caspases [4] that act on many substrates, including cytoskeletal molecules [5–9].

Previous studies have shown the mechanisms involved in the morphological changes occurring during the apoptotic death of neurons and how these changes contribute to the overall progression of apoptosis in neurons [2, 4, 10]. In cerebellar and hippocampal neurons, apoptotic cell death has been associated with cytoskeletal disruption [9, 11–17]. In some studies, cytoskeletal disruptors such as nocodazole promote apoptosis of neuronal cells, suggesting that cytoskeletal alteration could be a signal during the initial phases of apoptosis [13–17]. We have previously shown in cerebellar neurons that cytoskeletal proteins undergo a differential reorganization depending on the apoptotic condition [8]; also, cytoskeletal breakdown has been shown to be involved in neuronal apoptosis [9]. Although the morphological changes related to apoptosis are documented in neurons, the precise mechanisms involving the cytoskeletal changes during the progression of apoptosis are not well explored in astrocytes. In these cells, the actin cytoskeleton is known to play a role in the regulation of a variety of cellular actions such as cell attachment, motility, and morphological changes [18].

It is known that staurosporine (St) induces apoptosis in several cell types, including cerebellar astrocytes [19], a condition that involves changes in cell morphology from a flat to a stellate shape. Staurosporine is a competitive inhibitor of protein kinases that binds to kinases with high affinity and little selectivity [20]. This alkaloid has been demonstrated to inhibit cell cycle in different cell lines [21]. It also induces cell differentiation [22] and morphological changes in apoptotic cardiomyocytes [23] and hippocampal neurons [24]. Staurosporine has been considered a valuable tool for the study of apoptosis [25]. Its mechanisms of action include the activation of caspases through JNK1 and AP-1 activation in cell lines [26] or the p38 pathway activation in cerebellar granule neurons [27].

On the other hand, a large body of evidence has shown that during the process of neuronal death a generation of reactive oxygen species (ROS) [28–31] occurs. Moreover, it has been demonstrated that antioxidants prevent cell death, suggesting a key role of ROS in the death process. In cultured neurons, ROS have been shown to act as early signaling molecules in the death of cerebellar neurons induced by St or potassium deprivation [28, 29]. During spinal cord development, the physiological elimination of motoneurons is also regulated by ROS [30]. In addition, the redox regulation of several members of the MAPK pathway has been shown to be critical in the cell death mechanisms [27, 31, 32].

Although ROS can be generated by several sources, it has been suggested that NADPH oxidase (NOX) could be critical in the production of ROS involved in cell death. It is known that NOX has several homologues termed NOX 1 to 5 and DUOX 1 and 2, which are widely distributed in vertebrate tissues, including the nervous system [29, 33–38]. NOX2 was originally described in phagocytic cells, where it is responsible for the respiratory burst [39]. Many studies have shown that the inhibition of NOX activity remarkably protects neurons from cell death; however, in contrast to neurons, studies about the participation of ROS and NOX in the cell death of astrocytes are limited. In a previous study, NOX was shown to be the source for ROS involved in cell death and astrocyte survival [40]. Astrocytes exposed to acute H_2_O_2_ increased ROS levels, decreased glutamate uptake, and underwent oxidative damage and cell death [41]. The role of the oxidation of cytoskeletal proteins during the death of astrocytes is still not well understood.

Cytoskeleton alteration plays a key role in the regulation of diverse physiological processes. This alteration implies changes in the expression and/or reorganization of proteins such as actin, tubulin, and the proteins associated with the cytoskeletal proteins. On the other hand, ROS, particularly those produced by a NOX, have been implicated in the regulation of cytoskeletal remodeling [42]. For example, the oxidation of the actin binding protein cofilin stimulates apoptosis [43] and impairs cytoskeletal function in T cells [44]. Also, a redox imbalance modifies the actin cytoskeleton of cortical astrocytes [45] and H_2_O_2_ alters astrocyte cytoskeleton through the activation of the p38 MAPK pathway [46].

In the present study, we assessed the participation of ROS in the death of cultured rat cerebellar astrocytes using St as death inducer. Specifically, we examined the ROS generation and NOX activity induced by St and the involvement of ROS generated by NOX in the astrocytic death induced by St. We also studied the possible participation of ROS and a NOX in the alteration of the morphology and cytoskeletal proteins of astrocytes treated with St.

## 2. Materials and Methods

### 2.1. Chemicals and Materials

Fetal calf serum, penicillin/streptomycin, and Trizol reagent were from GIBCO (Grand Island, NY, USA). Poly-L-lysine (mol. wt. > 300,000), trypsin, DNAse, MTT (3-(4,5-dimethylthiazol-2-yl)-2,5-diphenyltetrazolium bromide), propidium iodide (PI), cytochrome c, staurosporine, and 4-(2-aminoethyl)-benzenesulfonyl fluoride (AEBSF) were from Sigma (St. Louis, MO, USA). Dihydroethidium and calcein-AM were purchased from Molecular Probes, Invitrogen (Carlsbad, CA, USA). Mouse anti-actin monoclonal antibodies were from Chemicon Int., anti-*β*-tubulin monoclonal antibodies were from Sigma-Aldrich, and FITC-goat anti-mouse IgG conjugated were from Zymed Laboratories Inc. All other chemicals were of the purest grade available from regular commercial sources. The colonies of NOX2 (gp91phox) knockout mice and NOX3 knockout mice on a C57BL6 background were purchased from the Jackson Laboratory (Bar Harbor, ME, USA) and were bred in the animal house of our institute.

### 2.2. Cell Cultures

All animals used for this experimentation were treated in accordance with the accepted standards of animal care and the procedures were approved by the Local Committee of Research and Ethics of the Instituto de Fisiología Celular, Universidad Nacional Autónoma de México. The protocol used followed the Guidelines for the Care and Use of Mammals in Neuroscience and the guidelines released by the Mexican Institutes of Health Research and the National Institutes of Health guide for the care and use of laboratory animals. All efforts were made to minimize animal suffering and to reduce the number of animals used.

Astrocytes were obtained from 8-day-old Wistar rats and 7-day-old NOX2^−/−^, NOX3^−/−^, or wild type mice as previously described by Moran and Patel [47]. Briefly, the dissociated cells suspensions from rat or mouse cerebella were plated at a density of 1 × 10^6^ cells/cm^2^. The culture medium consisted of basal Eagle's medium supplemented with 10% heat-inactivated fetal calf serum, 8 mM glucose, 2 mM glutamine, 50 U/mL penicillin, and 50 *μ*g/mL streptomycin. The culture dishes were incubated at 37°C in a humidified 5% CO_2_/95% air atmosphere. After 2 weeks in culture, cells were pretreated with antioxidants or NADPH oxidase inhibitors for 1 h and then treated with St (500 nM) for the indicated times and cells were used for different purposes. For immunofluorescence assays, cells were cultured on cover slips as indicated above. Morphology of astrocytes was evaluated by observations under a phase contrast microscope coupled to a digital camera.

### 2.3. Cell Viability

Cell viability was estimated by using calcein AM and propidium iodide (PI) to identify living and dead cells, respectively. Calcein enters viable cells and emits green fluorescence and when it is cleaved by esterases it can no longer permeate cell membranes. PI is not permeable to cell membranes and only dying cells are stained. After treatment, cells were incubated with calcein (1 *μ*M) for 15 min and with PI (40 *μ*M) for 5 min at 37°C and cells were observed in an epifluorescence microscope, using an excitation wavelength of 485–520 nm for calcein and 450–510 nm wavelengths for PI and the number of labeled cells with calcein and PI were counted.

Cell viability was also estimated at 6, 12, and 24 h after treatment by the conversion of 3-(4,5-dimethylthiazol-2-yl)-diphenyltetrazolium bromide (MTT) to formazan blue. MTT (0.25 mg/mL) was added to astrocyte cultures and incubated for 10 min at 37°C in 5% CO_2_-95% air atmosphere. After removal of the medium, 100% DMSO was added to the dishes and formazan blue formed was quantified spectroscopically at 570 nm excitation wavelength. A correspondence between the ability of cells to form formazan blue and the number of cells and DNA content [48] has been shown.

### 2.4. RT-PCR

Total RNA was isolated from cultured astrocytes with the single-step method based on guanidine isothiocyanate/phenol/chloroform extraction using Trizol reagent (Gibco-BRL). RNA concentration was determined by absorbance at 260 nm and its integrity was verified by electrophoresis on 1.1% denaturing agarose gels in the presence of 2.2 M formaldehyde. Total RNA was reverse transcribed to synthesize single strand cDNA. Four microliters of RT reaction was subjected to PCR in order to amplify NOX1, NOX2, and NOX4. The sequences of the primers for NOX1 were 5′-[CCT TCT GGG AAA CCT GCC TTT AG]-3′ in the sense primer and 5′-[TGT TGG TCA CAC TGG ATG ATA AGC]-3′ in the antisense; to NOX2 they were 5′-[TGG AGT GGT GTG TGA ATG CCA GAG]-3′ in the sense primer and 5′-[CGG ACA GCG ACT GCT GA]-3′ in the antisense; and to NOX4 they were 5′-[AGC CAA GAT TCT GAG ATT CTG CC]-3′ in the sense primer and 5′-[CCG AGG ACG CCC AAT AAA AAG]-3′ in the antisense. Forty-five microliters of PCR products was separated in 1.5% agarose gel and was stained with ethidium bromide. The image was captured under a UV transilluminator with a Type 665 negative film (Polaroid Co). The intensity of bands was quantified by densitometry using the Image J Program (NIH Image version 1.38x).

### 2.5. ROS Levels

ROS levels were measured with dihydroethidine (DHE). Cells were plated on coverslips and after treatments cells were incubated for 30 min with 1 *μ*M DHE in culture medium at 37°C. After incubation, cells were washed three times with PBS, fixed with 3% formaldehyde at 4°C for 15 minutes, washed with PBS, and mounted with PBS-glycerol 1 : 1 v/v. Preparations were observed under an epifluorescence microscope (Nikon Diaphot TMD, Nikon Corp., Japan) using a rhodamine filter (excitation filter wavelength/dichromatic mirror cut-on wavelength/barrier filter wavelengths of 510–560/565/590 nm). Results are expressed as mean fluorescence intensity.

### 2.6. NADPH Oxidase (NOX) Activity

NOX activity was evaluated as superoxide produced by NOX, which was measured in a quantitative kinetic assay based on the SOD-inhibitable reduction of cytochrome c [29]. Cultured cells were homogenized in a saline buffer (150 mM K, Na-phosphate, pH 7.4 supplemented with 1 mM MgCl_2_, 1 mM EGTA, and 2 mM NaN_3_). Cell homogenates were incubated with a reaction mixture containing 65 mM K, Na-phosphate, pH 6.8, 1 mM MgCl_2_, 1 mM EGTA, 2 mM NaN_3_, 0.1 mM cytochrome c, 10 mM FAD, and 0.1 mM SDS. The reaction started with the addition of 0.2 mM NADPH to the reaction mixture. The reference cuvette additionally contained 300 U/mL SOD. Reduction of cytochrome c was measured at 550 nm. Results are expressed as the difference between reduced and oxidized cytochrome c per hour per milligram of protein.

### 2.7. Immunofluorescence

Astrocytes were grown on poly-L-lysine-coated glass slides and treated with St (0.5 *μ*M) for different periods of time to induce cell death. Cells were immediately fixed with 4% paraformaldehyde for 20 min. After blocking with PBS (containing 1% BSA), the samples were incubated with mouse anti-actin (1 : 100 dilution), anti-*β*-tubulin (1 : 200 dilution), or anti-*α*-tubulin (1 : 200 dilution, Sigma-Aldrich, cat.T6199) monoclonal antibodies. Primary antibodies were incubated for 1 h at room temperature, followed by 1 h incubation with FITC-goat anti-mouse IgG conjugated (1 : 250 dilution) at room temperature. Coverslips were mounted using Vectashield mounting media with DAPI (Vector Labs). Stained cells were visualized under an epifluorescence microscope using a 40x objective (Nikon Diaphot-TMD) and their digitalized fluorescence images were captured.

### 2.8. Statistical Analysis

Data are presented as mean ± SEM and statistical significance of the results was determined by one-way analysis of variance (ANOVA) followed by Bonferroni's test. The statistical significance in the comparisons between wild type and NOX2 KO/NOX3 KO mice was determined by Student's *t*-test. *P* values less than 0.05 were considered statistically significant. Statistical significance of data from Figure 2(a) was determined by a nonparametric analysis followed by Dunnett's post hoc test.

## 3. Results

### 3.1. Staurosporine Induces Death of Astrocytes

**Figure 1:**
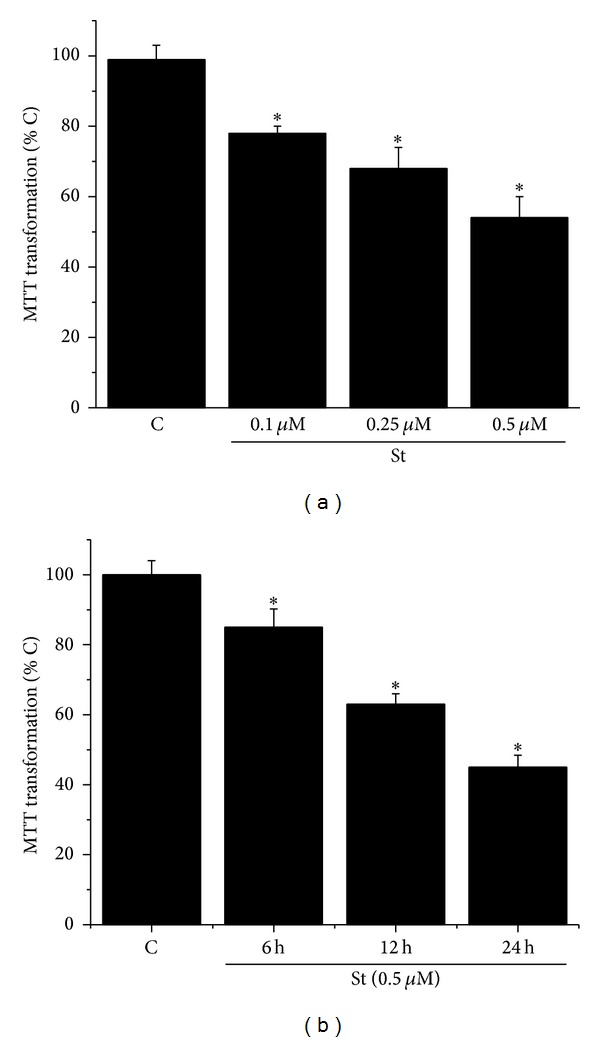
Staurosporine reduces cerebellar astrocytes viability. (a) Cerebellar astrocytes were treated with staurosporine 0.1 *μ*M, 0.25 *μ*M, and 0.5 *μ*M for 24 h and the cell viability was measured by MTT transformation as detailed in Section 2. (b) Cerebellar astrocytes were treated with staurosporine 0.5 *μ*M for 6, 12, and 24 h and the cell viability was measured by MTT transformation as detailed in Section 2. Data are presented as mean ± SEM of four independent experiments. ∗ is significantly different from control (*P* < 0.05).

**Figure 2:**
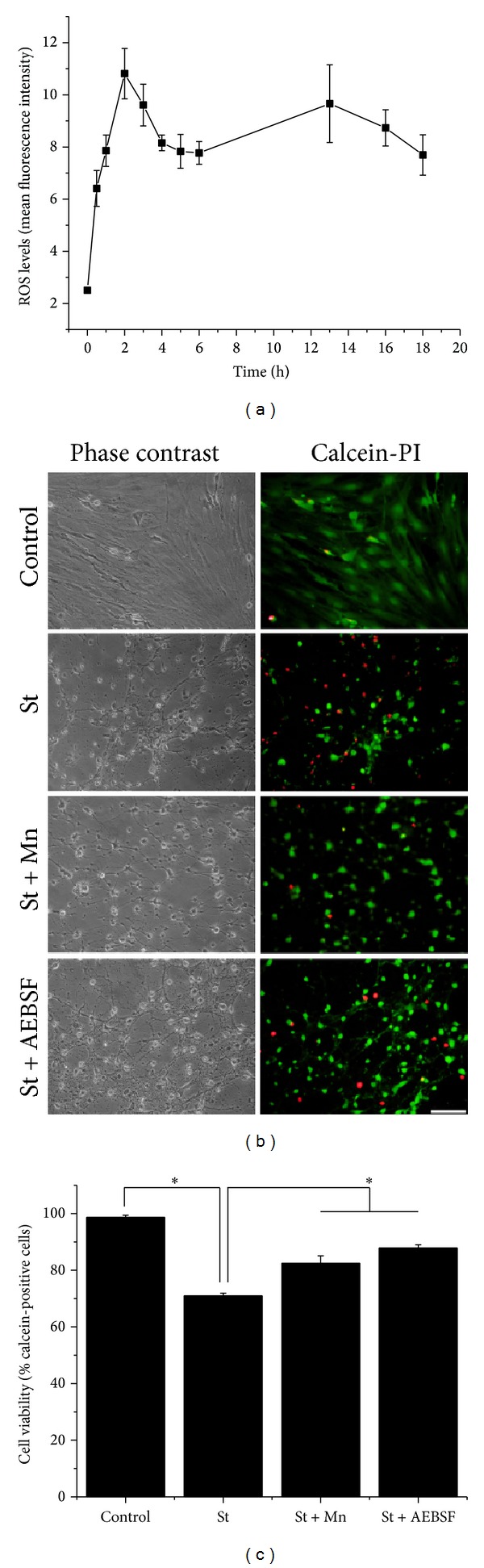
Reactive oxygen species are involved in cerebellar astrocytes death induced by St. (a) The levels of ROS were measured during different times by the oxidation of dihydroethidium (1 *μ*M) as detailed in Section 2. Data are presented as mean ± SEM of four independent experiments. All points are significantly different from control (0 h) (*P* < 0.001). (b) Representative micrographs of cerebellar astrocytes treated with St (0.5 *μ*M) for 24 in the presence or absence of MnTMPyP (50 *μ*M) or AEBSF (50 *μ*M). Cells were marked with calcein (green) and propidium iodide (red). Scale bar represents 50 *μ*m. (c) Cell viability was determined by the percentage of calcein-positive cells from the total number of cells (calcein-positive cells plus propidium iodide-positive cells). Data are presented as mean ± SEM of three independent experiments.

Figure 1 shows that St induces a reduction in cell viability of astrocytes, measured as the MTT transformation, in a time- and concentration-dependent manner. Treatment with St during 24 h induced a 22% reduction of cell viability at a concentration of 0.1 *μ*M and by 55% at 0.5 *μ*M (Figure 1(a)). When astrocytes were subjected to 0.5 *μ*M St treatment, cell viability was decreased by 40% and 55% at 12 and 24 h, respectively (Figure 1(b)). These results were supported by the morphological appearance of astrocytes observed by phase contrast microscopy (Figures 2(b) and 6).

### 3.2. St Induced Cell Death Is Dependent on ROS Production

It was previously reported [27–32] that some apoptotic conditions induce the generation of ROS in neuronal cells. Therefore, we evaluated the ROS production in astrocytes treated with St (0.5 *μ*M). Figure 2(a) shows that St induced ROS generation, which were measured as dihydroethidium- (DHE-) derived fluorescence (fluorescence intensity). ROS levels increased after 1 h of treatment with St, reaching a maximal level at 2 h. At this time, ROS decreased and from 6 h to 18 h ROS levels remained relatively high without change.

In order to evaluate the roles of ROS and NOX in the death of astrocytes induced by St, we tested the effects of the antioxidant MnTMPyP and the nonspecific inhibitor of NOX (Figures 2(b) and 2(c)). Under these conditions, both, the antioxidant and the NOX inhibitor AEBSF, significantly reduced cell death by 45%. Similarly, the presence of the NOX inhibitor markedly inhibited cell death by 65% (Figure 2(c)). These results were confirmed by the morphological changes of the astrocytes observed in the phase contrast microscopy (Figure 2(b)).

### 3.3. Astrocytes Express NOX Subunits and St Induces NOX Activity

**Figure 3:**
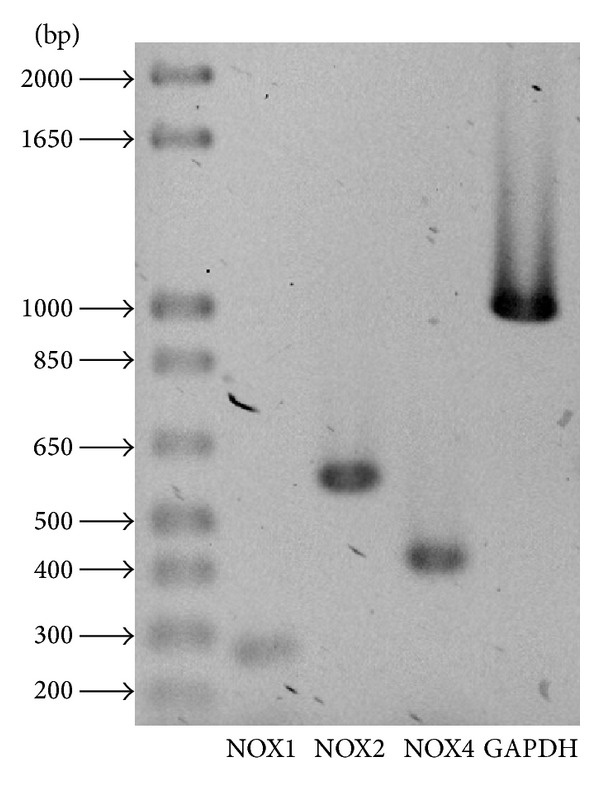
NOX subunits are expressed in cerebellar astrocytes. NOX subunits expression was determined by RT-PCR assays in cultured astrocytes as detailed in Section 2 for NOX1 (268 bp), NOX2 (558 bp), and NOX4 (408 bp). Three independent assays were performed.

**Figure 4:**
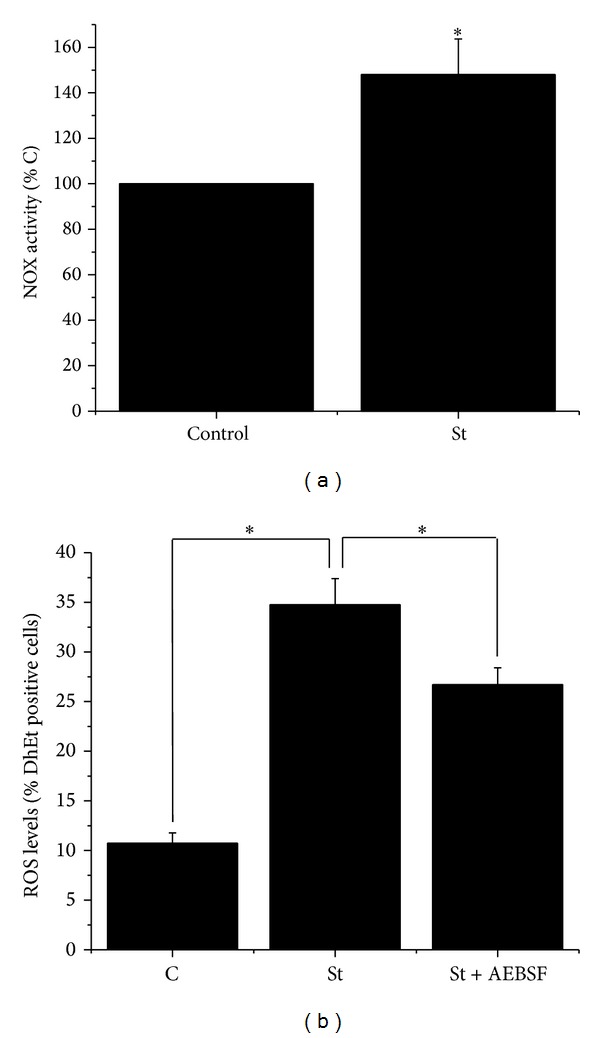
Staurosporine induces NOX activity. (a) NOX activity was evaluated at 2 h as detailed in Section 2. Data are presented as mean ± SEM of five independent experiments. ∗ is significantly different from control (*P* < 0.05). (b) ROS levels were determined in cerebellar astrocytes treated with St (0.5 *μ*M) for 2 h in the presence or absence of AEBSF (50 *μ*M) by measuring the oxidation of dihydroethidium (1 *μ*M) as detailed in Section 2. Data are presented as mean ± SEM of three independent experiments.

The expression of NOX was evaluated by RT-PCR analysis in astrocytes by using specific oligonucleotides for NOX1, NOX2, and NOX4.  Figure 3 shows that astrocytes cultured for 2 weeks expressed mRNA of all three NOX homologues tested. We also measured the NOX activity induced by St in astrocytes after 2 h of treatment, when the maximal production of ROS was observed. Under these conditions, NOX activity was increased by 50% (Figure 4(a)). In addition, the observed increase of ROS production induced by St was partially inhibited by the NOX inhibitor AEBSF (Figure 4(b)).

### 3.4. Cell Death Induced by St Is Not Reduced in NOX2- and NOX3-Deficient Astrocytes

**Figure 5:**
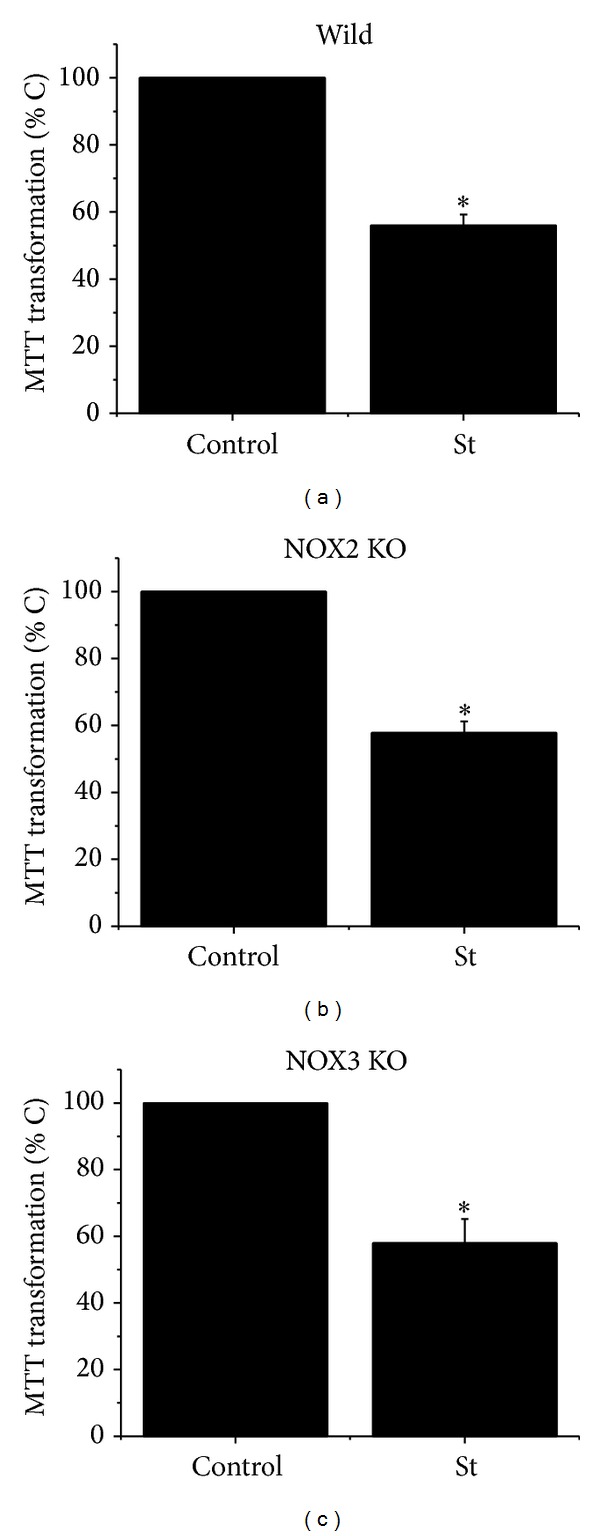
Cell death induced by St is not mediated by NOX2 and NOX3. Cerebellar astrocytes were obtained from wild type mice and NOX2 KO and NOX3 KO mice. Cells were treated with St (0.5 *μ*M) for 24 h and the cell viability was estimated as MTT transformation as detailed in Section 2. Data are presented as mean ± SEM of five independent experiments. ∗ is significantly different from control (*P* < 0.05).

In order to evaluate the role of specific NOX homologues in the death induced by St in cerebellar astrocytes, we tested the effect of St in cultured astrocytes obtained from NOX2 KO and NOX3 KO mice as described in Section 2.  Figure 5 shows that St induced more than 50% cell death after 24 h of St treatment in wild type astrocytes. Similar results were found when cell viability was measured in NOX2 KO and NOX3 KO astrocytes treated with St.

### 3.5. Rearrangement of the Cytoskeleton during the Cell Death Induced by St

**Figure 6:**
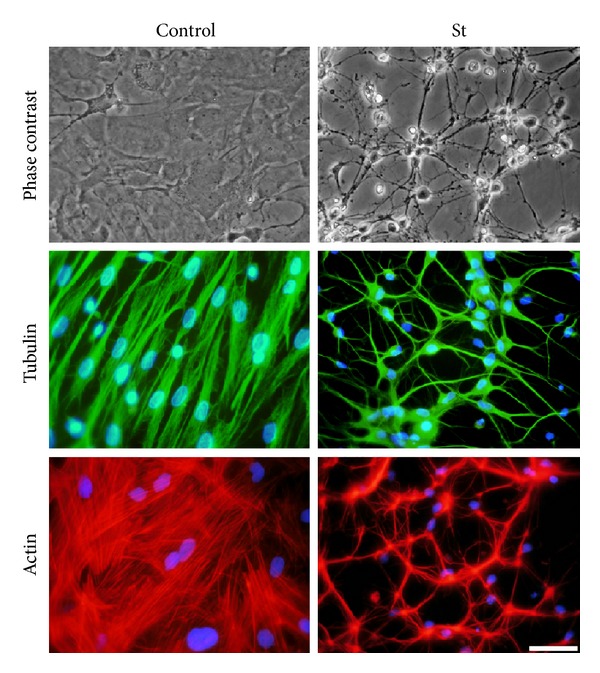
Morphological changes of astrocytes induced by St are evidenced by the rearrangement of cytoskeletal proteins. Astrocytes were treated with St (0.5 *μ*M) for 12 hours and then were labelled with rhodamine-phalloidin or immunostained for tubulin as detailed in Section 2. Representative images of phase contrast, rhodamine-phalloidin, and tubulin are shown in control and St treated astrocytes. Scale bar represents 50 *μ*m.

Astrocytes showed morphological changes during cell death induced by St. After 12 h of St treatment cells showed somatic shrinkage and the formation of neurite-like processes with an apparent reticular aspect (Figure 6) that eventually fragmented and practically disappeared after 24 h (not shown). In order to explore the relationship between cytoskeleton and the morphological changes induced by St described above, we studied the immunofluorescent patterns of actin and tubulin organization after St treatment. Figure 6 shows that, under control conditions, actin, *α*-tubulin, and *β*-tubulin (Figure 8) were homogeneously distributed in the soma and organized in fibers running longitudinally. After 12 h of St treatment, these proteins markedly compacted following the morphological changes described above. Particularly, *α*-tubulin was equally distributed in both soma and processes, but actin showed a tendency to be located in the processes (Figure 6).

**Figure 7:**
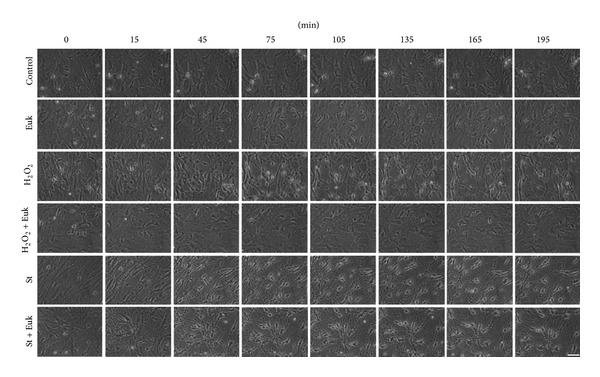
Effect of St and hydrogen peroxide on the morphology of cerebellar astrocytes. Time-lapse images of astrocytes pretreated for 2 h with Euk-134 (20 *μ*M) in the presence or absence of hydrogen peroxide (200 *μ*M) and St (0.5 *μ*M) were taken from the same field. Scale bar represents 50 *μ*M.

In order to evaluate the role of ROS in the morphology and cytoskeleton rearrangement, we first studied the action of a prooxidant condition in the astrocytic morphology.  Figure 7 shows that the presence of 200 *μ*M H_2_O_2_ induced morphological changes after 45 min of treatment. The observed changes in the cell morphology were similar to those observed in astrocytes treated with St, that is, cell body shrinkage and formation of thin processes. The observed changes in astrocytes were evident after 15 min of treatment (Figure 7). Although the prooxidant condition also induced a cell shrinkage, the formation of process-like extensions was less evident and they did not form the reticular structure observed with St. More importantly, astrocytes treated simultaneously with the antioxidants EUK and H_2_O_2_ did not show any morphological change. In contrast, the treatment with the antioxidants EUK did not affect the changes induced by St (Figure 7).

**Figure 8:**
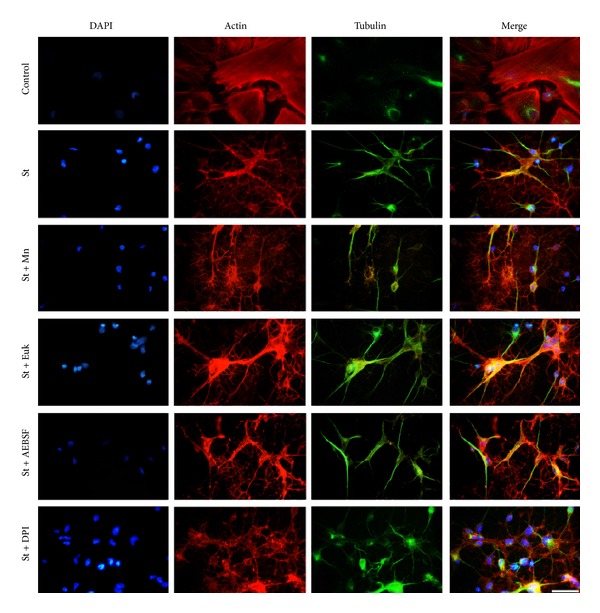
Cytoskeletal rearrangements induced by St are not mediated by ROS. Cerebellar astrocytes were treated with St (0.5 *μ*M) for 2 h in the presence or absence of the antioxidants MnTMPyP (50 *μ*M) and Euk-134 (20 *μ*M) and the NOX inhibitors DPI (520 nm) and AEBSF (50 *μ*M). Immunostaining against *β*-tubulin (green) and staining with rhodamine-phalloidin (red) were performed as mentioned in Section 2. Scale bar represents 50 *μ*M.

In order to further examine the participation of ROS in the cytoskeletal organization, under the apoptotic conditions previously described, we evaluated the effect of antioxidants and NOX inhibitors on the organization of actin and tubulin in astrocytes. Under these conditions, we found that neither the antioxidants MnTPMyP and EUK nor the NOX inhibitors DPI and AEBSF induced any significant change in the rearrangement of *α*-tubulin or actin induced by St (Figure 8).

### 3.6. Expression of Tubulin and Actin during Apoptotic Death of Astrocytes Induced by St

**Figure 9:**
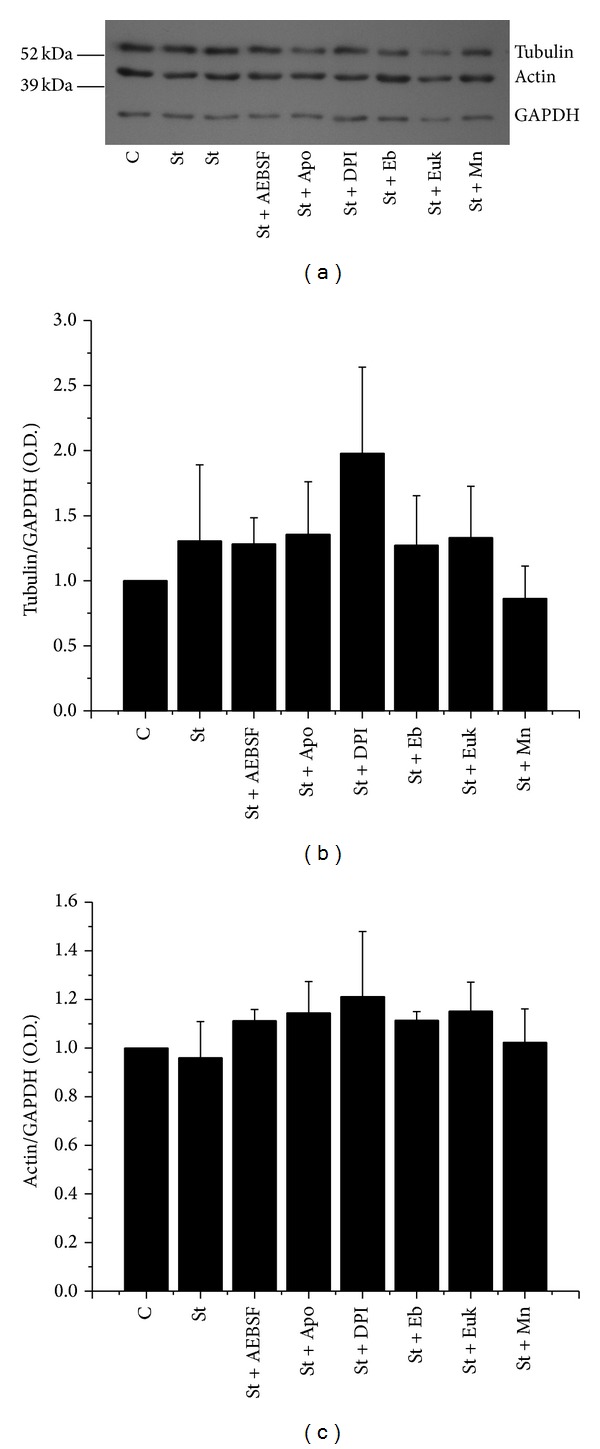
St does not induce changes in the expression of cytoskeletal proteins. Actin and *α*-tubulin levels were determined by Western blot assays as detailed in Section 2. Cerebellar astrocytes were treated with St (0.5 *μ*M) in the presence or absence of the antioxidants MnTMPyP (50 *μ*M) and Euk-134 (20 *μ*M) and the NOX inhibitors DPI (520 nm) and AEBSF (50 *μ*M). Data are presented as mean ± SEM of three independent experiments. No statistical differences were found among the treatments.

We evaluated by Western blot analysis the effect of St on the levels of actin and *α*-tubulin in astrocytes. We found that this condition did not change neither *α*-tubulin nor actin expression levels (Figure 9(a)). On the other hand, we also found that any antioxidant or NOX inhibitor tested induced changes in the protein levels of these two cytoskeletal proteins (Figure 9(a)). Densitometric analysis shows that neither St nor the antioxidants, EUK, MnTPMyP, and Ebselen, nor the NOX inhibitors, AEBSF, apocynin, and DPI, had any effect in the observed expression of *α*-tubulin (Figure 9(b)) and actin (Figure 9(c)).

## 4. Discussion

A large body of evidence has shown that ROS regulate cell death under pathological and physiological conditions. In cerebellar neurons, apoptotic death involves ROS generation [27–29, 31] that plays a central role in the initiation and execution of neuronal apoptosis [28, 49–51], probably acting as an early signal. On the other hand, we also demonstrated an active role of NOX in the apoptotic death of cerebellar neurons [29]. Although ROS has been proposed to play a central role in neurons, there is a lack of information about the action of ROS in the apoptotic death of astrocytes. We, therefore, evaluated the role of ROS and the possible participation of NOX as a source of ROS in the death of cerebellar astrocytes and their involvement in the morphological and cytoskeletal changes associated with this process.

Cultured astrocytes have been shown to be an appropriate model for the study of cell death [52]. Also, St has been considered a useful tool to induce apoptosis [25]. In particular, there are several reports showing that St induces apoptotic death of astroglial cells in culture [53–57]. In the present study, we found in cerebellar astrocytes that St induced cell death in a time- and concentration-dependent manner. We also found that St treatment induced a differential ROS generation. After 1 h of treatment, it was observed that there was a significant increase of ROS and the highest ROS levels were detected after 2 h of treatment. This result coincides with previous observations in other preparations showing a transient ROS production before the activation of the cell death [28, 50, 51, 58], which suggests that ROS could be involved in the process of cell death in astrocytes. The possibility that ROS could be required for the initiation and/or progression of cell death in these cells was further supported by the fact that the antioxidant MnTMPyP was able to rescue astrocytes from cell death induced by St. Interestingly, a nonspecific NOX inhibitor, AEBSF, was also able to significantly reduce cell death induced by St. These results suggest that astroglial cell death induced by St could be mediated, at least partially, by ROS produced by a NOX.

It is well accepted that NOX participates in several physiological and pathological processes in a wide variety of cell types. The only described function of NOX is to generate O_2_^•−^ that can be converted to other ROS. It has been proposed that ROS generated by this complex could mediate cell death [27, 29, 31, 33, 34]. Several studies have shown extensive expression of the NOX homologues in several tissues [29, 33–35, 39]. However, not much information is available about the expression of NOX homologues in astrocytes. It was originally suggested, by indirect evidence, that NOX was present in cortical astrocytes [59]. More recently, the presence of NOX1, NOX2, NOX4, and Duox 1 in astrocytes from cerebral cortex [60] was shown. It is also known that NOX5 is not present in murines [33, 34, 61]. In this study, we show for the first time that NOX1, 2, and 4 are present in primary cultures of cerebellar astrocytes.

There are some reports suggesting that conditions that induce death of astrocytes involve the participation of NOX [62, 63]. In this study, the participation of a NOX in the cell death process induced by St was supported by the observation that this condition increases NOX activity after 2 h of St treatment. At this time, the transient ROS production induced by St was maximal. Moreover, ROS levels induced by St were significantly reduced in the presence of the NOX inhibitor AEBSF. Finally, we found in mice deficient in NOX2 or NOX3 that the cell viability of astrocytes treated with St was not significantly different from astrocytes from wild type mice. This suggests that NOX other than these two homologues could be responsible for the generation of the ROS implicated in the death of astrocytes. In a previous study, we found that ROS produced by NOX2 were responsible for the death of cerebellar neurons induced by St, but not by potassium deprivation. In this regard, a possible source for ROS involved in astrocytic death by St could be NOX1 or NOX4, which was expressed in cerebellar astrocytes. An alternative explanation for the lack of effect of NOX2 or NOX3 deficiency in death induced by St is a possible compensation phenomenon that has been reported for these complexes in other preparations [64].

Apoptotic cell death is characterized by a series of morphological changes. Some of the morphological features observed during apoptosis include nuclear condensation, cell shrinkage, and retraction of cellular processes [3]. A large body of evidence suggests that the cytoskeleton is critical in the morphological changes during apoptotic progression induced in different cell death models [5, 6]; however, no information exists about the particular changes of the cytoskeletal proteins during cell death of astrocytes induced by St treatment.

In this study, we showed that St induces morphological alterations in astrocytes during the first minutes of treatment. These morphological changes could be due to a differential reorganization of cytoskeletal proteins such as actin and tubulin induced by cell death conditions. In this regard, some studies have established that during cell death there is rearrangement and accumulation of cytoskeletal proteins including actin and tubulin [65, 66]. This could be accompanied by the ability of microtubules to have spontaneous changes in the polymerization and depolymerization activities during apoptosis [67].

We observed a correlation between the morphological alterations and the cytoskeleton changes induced by the apoptotic condition. Both actin and tubulin (*α*-tubulin and *β*-tubulin) showed changes in astrocytes treated with St. This could be as a result of a dynamic behavior of actin and tubulin in apoptosis during the dismantling of the cell before the complete loss of cell viability [65]. Under these conditions, fibers of stress that determine the flattened or polygonal morphology lost their arrangement, forming small clusters. Also, the actin filaments were organized in beams. In the case of the microtubules, St treatment induced their rearrangement as diffuse networks that arise in concentrated points.

It is known that the organization of the actin cytoskeleton is a key element in the morphology of astrocytes [68] and that members of the Rho family regulate their arrangement [69–71]. The morphological changes associated with St could be mediated by an inhibition of Rho. It has been shown that the activity of these kinases promote the depolymerization of stress fibers, inducing the morphological change toward a stellate shape [72]. In addition, the activation of Rho stabilizes subpopulations of microtubules [73]. If the Rho pathway is inactivated, stress fibers are depolymerized and consequently a morphological change occurs. It is noteworthy to mention that no changes in the levels of actin and *α*-tubulin were observed at early times after St treatment, suggesting that the observed morphological alterations were due to a rearrangement of the cytoskeleton, but not due to a change in the level of expression of these proteins.

Considering that ROS could be directly or indirectly responsible for the observed morphological changes as part of the death process, we evaluated the possible involvement of ROS in this event. This idea was supported by the fact that H_2_O_2_ treatment induced clear morphological changes in astrocytes at early times, similarly to what was observed with St treatment. In addition, the observed changes in the morphology of astrocytes induced by St occurred early in time as it occurred for ROS production with St. However, the first noticeable morphological alterations induced by St (i.e., 15 min) (data not shown) happened at a time when ROS levels had not increased. More conclusive was the observation that the presence of an antioxidant in the cultures did not inhibit the morphological changes induced by St, in contrast to what was observed in astrocytes treated with H_2_O_2_. Similarly, the presence of a NOX inhibitor also failed to prevent the morphological changes induced by St. These results strongly suggest that neither NOX nor ROS seem to be implicated in the morphological changes induced by St. The above conclusion was further supported by the observation that both treatments with antioxidants and NOX inhibitors did not modify the described reorganization of actin and tubulin induced by St. The levels of these two proteins, which were not altered by St, were also unaffected by antioxidants.

In conclusion, we found here that ROS play a role in the death of astrocytes induced by staurosporine and that a possible source for these ROS is a member of the NOX family. We also found that NOX1, NOX2, and NOX4 are present in astrocytes, but only NOX1 and/or NOX4 could have a role in cell death of astrocytes induced by St. Finally, we showed that the actin and tubulin proteins suffer modifications related to the observed morphological changes in astrocytes treated with St, but these modifications are not dependent on NOX or ROS produced by St treatment. Overall, these results confirm the idea that NOX could contribute to death of cerebellar astrocytes.
